# A case report of glecaprevir/pibrentasvir-induced severe hyperbilirubinemia in a patient with compensated liver cirrhosis

**DOI:** 10.1097/MD.0000000000017343

**Published:** 2019-09-27

**Authors:** Jae Hyun Yoon, Sun Min Kim, Gaeun Kang, Hee Joon Kim, Chung Hwan Jun, Sung Kyu Choi

**Affiliations:** a Department of Gastroenterology and Hepatology; b Department of Clinical Pharmacology; c Department of Surgery, Chonnam National University Hospital and School of Medicine, Gwangju, South Korea.

**Keywords:** cirrhosis, elderly, glecaprevir, hepatitis C virus, hyperbilirubinemia

## Abstract

**Rationale:**

Glecaprevir/pibrentasvir, a pan-genotypic and ribavirin-free direct acting antiviral agent regimen, has shown significant efficacy and very few serious complications. However, as the drug metabolizes in the liver, it is not recommended in patients with decompensated liver cirrhosis. Herein, we report the case of a patient with compensated liver cirrhosis who developed severe jaundice after glecaprevir/pibrentasvir medication.

**Patient concerns:**

A 77-year-old man diagnosed with chronic hepatitis C-related compensated liver cirrhosis visited hospital due to severe jaundice after 12 weeks of glecaprevir/pibrentasvir medication.

**Diagnoses:**

On the laboratory work-up, the total/direct bilirubin level was markedly elevated to 21.56/11.68 from 1.81 mg/dL; the alanine aminotransferase and aspartate aminotransferase levels were within the normal range. We checked the plasma drug concentration level of glecaprevir, and 18,500 ng/mL was detected, which was more than 15 times higher than the drug concentration level verified in normal healthy adults.

**Interventions:**

Glecaprevir/pibrentasvir was abruptly stopped and after 6 days, the drug concentration level decreased to 35 ng/mL and the serum total/direct bilirubin decreased to 7.49/4.06 mg/dL.

**Outcomes:**

Three months after drug cessation, the serum total bilirubin level normalized to 1.21 mg/dL and HCV RNA was not detected.

**Lessons:**

We report what is likely the first known case of severe jaundice after medication with glecaprevir/pibrentasvir in a patient with compensated liver cirrhosis. Clinicians should bear potential hyperbilirubinemia in mind when treating chronic hepatitis C with this regimen and should monitor the patient closely during follow-up laboratory exams, especially in elderly cirrhotic patients.

## 1 Introduction

With the introduction of direct acting antiviral agents (DAA), the efficacy and safety of chronic hepatitis C infection treatment have improved significantly.^[1]^ As ribavirin-free, pan-genotypic regimens have been recently developed, the treatment of hepatitis C virus (HCV) infection is more convenient, with fewer side effects. The glecaprevir plus pibrentasvir regimen is a ribavirin-free DAA that has the advantage of shorter duration compared with other regimens; moreover, it is a pan-genotypic agent recommended in all genotypes of HCV infection.^[2]^ However, glecaprevir, a nonstructural (NS) protein 3/4A protease inhibitor, is mainly eliminated through biliary excretion and is contraindicated in patients with moderate to severe hepatic impairment (Child–Turcotte–Pugh [CTP] score B or C) due to increased plasma drug concentration area under the curve (AUC).^[3]^

A similar dosage of glecaprevir/pibrentasvir (GP) is recommended for patients with chronic hepatitis C and compensated liver cirrhosis; there has been no report of serious hepatotoxicity so far. An integrated analysis of clinical trials with GP reported elevated total bilirubin levels of grade 3 or higher in 3 patients, but these elevations were transient and resolved without requiring discontinuation of the drug.^[4]^ Herein, we report what is, to the best of our knowledge, the first case of an elderly patient with compensated liver cirrhosis who developed severe jaundice after 3 weeks of the GP regimen.

## 2 Case presentation

The patient was a 77-year-old man with chronic hepatitis C-related compensated liver cirrhosis. He visited our institution to evaluate and manage a 2-cm sized enhancing liver mass and fibro-calcified densities in the lung parenchyma with interlobar effusion detected on chest and abdomen computed tomography. The liver mass was diagnosed as a hepatocellular carcinoma (HCC) with magnetic resonance imaging and was treated using radiofrequency ablation (RFA). Complete remission of HCC after RFA was maintained for more than 12 months. On sputum examination, *Mycobacterium tuberculosis* was cultured and pulmonary tuberculosis was diagnosed, due to which treatment with 1st regimen including isoniazid, rifampin, ethambutol, and pyrazinamide was started. After 1 month of anti-tuberculosis medication, hepatotoxicity was observed; the alanine aminotransferase (ALT) level was 53 U/L and the total bilirubin level was 6.51 ng/dL. Therefore, the regimen was switched to rifampin, ethambutol, and moxifloxacin. Due to potential drug–drug interactions between antituberculosis medication and direct-antiviral agents, treatment of the chronic hepatitis C infection was started 3 months after the pulmonary tuberculosis treatment ended.

The patient had underlying hypertension and benign prostate hyperplasia, for which he was on amlodipine plus valsartan and tamsulosin, respectively. Apart from this, the patient was not taking any other medications, including herbal agents.

**Figure 1:**
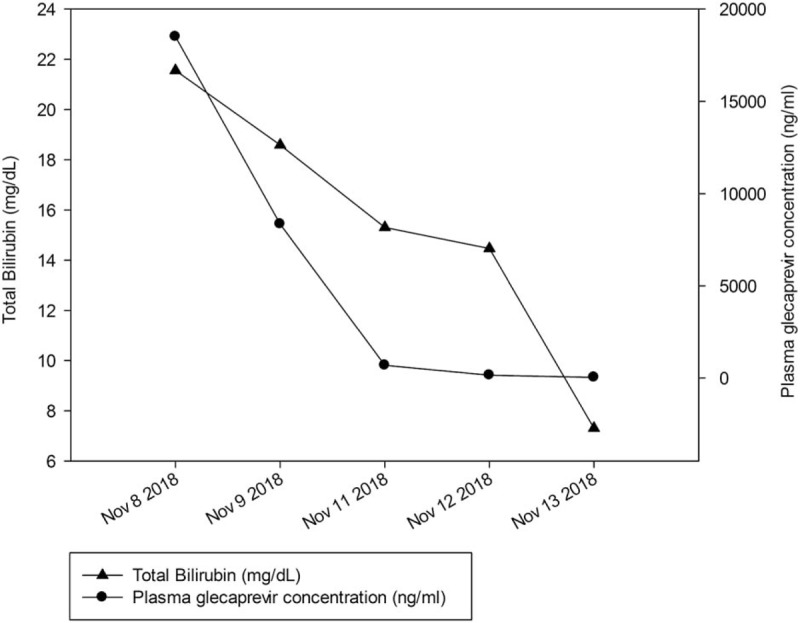
Course of plasma glecaprevir concentration and total bilirubin level.

The HCV genotype was confirmed as type 2a and we decided on treatment with the GP regimen (300 mg glecaprevir and 120 mg pibrentasvir) as many guidelines suggest.^[2,5,6]^ His baseline total bilirubin level was 1.81 mg/dL and the CTP score was 5, without esophageal or gastric varix on endoscopy; ascites was absent as well. After 3 weeks of GP medication, he visited our clinic due to pruritus, icteric sclera, and generalized weakness. He had body ache and weakness that had started the week after starting the medication. On laboratory examination, the total/direct bilirubin was markedly elevated to 21.6/11.7 mg/dL and ALT was within the normal range. As there was no history of recent ingestion of other drugs or alcohol, we considered grade 3 hepatotoxicity due to the GP regimen and abruptly stopped the medication. Six days after drug cessation, the total/direct bilirubin level decreased to 7.31/4.06 mg/dL. We checked the serum concentration level of glecaprevir with the rapid and selective liquid chromatography-tandem mass spectrometry method on the day of admission, the serum level was 18,500 ng/mL. After cessation of medication, as the drug concentration diminished, the total bilirubin level also decreased (Fig. 1). He recovered from body ache and fatigue after 9 days of admission and was discharged. We decided that treatment should not be reinitiated, and 2 months after discharge, the total bilirubin level was normalized to 1.21 mg/dL; 12 weeks after the end of treatment, HCV RNA was not detected.

In addition, the patient provided informed consent for the manuscript to be published.

## 3 Discussion and conclusions

To the best of our knowledge, this is the first report demonstrating severe hyperbilirubinemia induced by the GP regimen in a patient with chronic hepatitis C infection. NS3/4A protease inhibitors such as paritaprevir, grazoprevir, glecaprevir, or voxilaprevir are contraindicated in CPT B or C decompensated cirrhosis due to significantly elevated protease inhibitor concentrations and higher risk of liver toxicity.^[2]^ Despite possible hepatic toxicity, NS3/4A protease inhibitors are generally well tolerated in patients with CPT A compensated cirrhosis.^[7–9]^ Moreover, although cytochrome P450 (CYP) 3A, the main metabolizer for NS3/4A protease inhibitor, is known to be reduced in elderly patients, there are no reports of adverse events of N3/4A protease inhibitor in elderly patients.^[10–12]^

Many trials and studies have proved the high safety profile of GP regimen; especially, the EXPEDITION-1 trial, which studied the efficacy and safety of this regimen in cirrhosis patients, showed no complications of grade 3 adverse events in hepatotoxicity such as ALT, aspartate aminotransferase, or total bilirubin level elevation.^[13]^ The mean value of glecaprevir maximum plasma concentration level was reported as 1,150 to 1,390 ng/mL in normal healthy adult subjects.^[14]^ An analysis of previous phase 2 and 3 trials showed that plasma drug concentration levels were 2.2-fold higher in patients with cirrhosis than patients without cirrhosis.^[4]^ Even considering the higher drug concentration level in cirrhotic patients, our patient had about 13- to 16-fold higher plasma drug concentration level (18,500 ng/mL) when hyperbilirubinemia was diagnosed. Additionally, after cessation of the GP regimen, the plasma concentration level dropped rapidly and the total bilirubin level diminished as well. At the 2-months follow-up, the total bilirubin level was 1.21 mg/dL and there was no worsening of liver function; moreover, HCV RNA was not detected at 12 weeks, after treatment ended.

**Figure 2:**
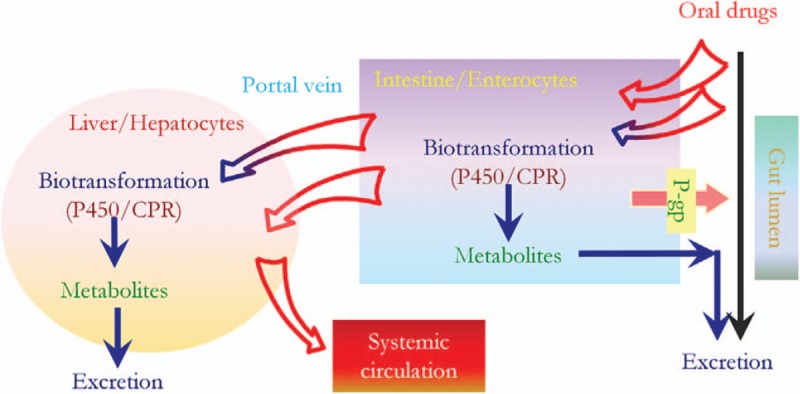
Sequential action of intestinal and hepatic P450 enzymes on orally ingested drugs. CPR = NADPH-cytochrome P450 reductase,^[15]^ P-gp = P-glycoprotein.

The patient had an adequate interval of more than 3 months between the antituberculosis medication and GP regimen, which potentially inhibits CYP3A. Additionally, he was not taking any drug including organic anion transporting polypeptide (OATP) 1B1/3, breast cancer resistance protein (BCRP), or p-glycoprotein inhibitors, all of which increase the plasma GP concentration. There were no clinical findings suggesting decompensated cirrhosis such as variceal bleeding or ascites. Considering the markedly elevated plasma glecaprevir concentration and no other drug interactions that might have influenced the drug metabolism, we assume that the high level of glecaprevir was derived from low activity of CYP3A, which is due to low reserves of liver function and old age (Fig. 2).^[15]^ Unfortunately, we could not check the patient's CYP3A level due to his poor general condition, but reduced CYP3A levels in elderly patients, and the correlation between grade of CYP3A reduction and worsening of liver function have been reported in several studies.^[10–12,16–18]^ Peculiarly, he had a history of intermittent mild hyperbilirubinemia (up to 4 mg/dL) while undergoing treatment for pulmonary tuberculosis even after switching tuberculosis medication and the hyperbilirubinemia resolved spontaneously without any intervention or admission. These events might have been the result of the patient's poor reserves of liver function, with deficiency of hepatic enzymes including CYP3A and multidrug resistance-associated protein (MRP) 2, which are necessary in rifampin metabolism.

This study has some limitations. First, we could not analyze CYP3A level which we assume to have had played a pivotal role in hyperbilirubinemia. Second, it is difficult to exactly explain the mechanism of HCV eradication with a very short DAA medication period.

Third, even though we are suggesting the most likely mechanism of unexpected hyperbilirubinemia, the actual exact mechanism of this adverse events needs to be verified with more case-series or scientific research. Further reports of large number of patients about hyperbilirubinemia in cirrhosis patients treated with GP regimen need to be followed.

In conclusion, we report the first known case of a patient with severe hyperbilirubinemia and compensated liver cirrhosis in whom glecaprevir plus pibrentasvir was used for treatment of chronic hepatitis C infection. Clinicians should be aware of possible severe hyperbilirubinemia when using the glecaprevir plus pibrentasvir regimen in elderly cirrhotic patients.

## Author contributions

**Conceptualization:** Chung Hwan Jun, Sung Kyu Choi.

**Data curation:** Gaeun Kang, Sun Min Kim, Hee Joon Kim.

**Formal analysis:** Gaeun Kang, Hee Joon Kim.

**Methodology:** Gaeun Kang.

**Writing – original draft:** Jae Hyun Yoon.

**Writing – review & editing:** Jae Hyun Yoon, Chung Hwan Jun, Sung Kyu Choi, Sun Min Kim, Hee Joon Kim.
